# Structure-Based Drug Design Studies Toward the Discovery of Novel Chalcone Derivatives as Potential Epidermal Growth Factor Receptor (EGFR) Inhibitors

**DOI:** 10.3390/molecules23123203

**Published:** 2018-12-05

**Authors:** Menier Al-Anazi, Belal O. Al-Najjar, Melati Khairuddean

**Affiliations:** 1 School of Chemical Sciences, Universiti Sains Malaysia, Penang 11800, Malaysia; mn.alenazi@ut.edu.sa; 2 Department of chemistry, Faculty of Science, University of Tabuk, Tabuk 71491, Kingdome of Saudi Arabia; 3 Faculty of Pharmacy and Medical Sciences, Al-Ahliyya Amman University, Amman 19328, Jordan; najjar.belal@gmail.com; 4 Molecular Modeling and Drug Design Lab, Al-Ahliyya Amman University, Amman 19328, Jordan

**Keywords:** anti-cancer, tyrosine kinase inhibitors, chalcone, molecular docking, molecular dynamics, MM-GBSA

## Abstract

Human Epidermal Growth Factor Receptor-1 (EGFR), a transmembrane tyrosine kinase receptor (RTK), has been associated with several types of cancer, including breast, lung, ovarian, and anal cancers. Thus, the receptor was targeted by a variety of therapeutic approaches for cancer treatments. A series of chalcone derivatives are among the most highly potent and selective inhibitors of EGFR described to date. A series of chalcone derivatives were proposed in this study to investigate the intermolecular interactions in the active site utilizing molecular docking and molecular dynamics simulations. After a careful analysis of docking results, compounds **1a** and **1d** were chosen for molecular dynamics simulation study. Extensive hydrogen bond analysis throughout 7 ns molecular dynamics simulation revealed the ability of compounds **1a** and **1d** to retain the essential interactions needed for the inhibition, especially MET 93. Finally, MM-GBSA calculations highlight on the capability of the ligands to bind strongly within the active site with binding energies of −44.04 and −56.6 kcal/mol for compounds **1a** and **1d**, respectively. Compound **1d** showed to have a close binding energy with TAK-285 (−66.17 kcal/mol), which indicates a high chance for compound **1d** to exhibit inhibitory activity, thus recommending to synthesis it to test its biological activity. It is anticipated that the findings reported here may provide very useful information for designing effective drugs for the treatment of EGFR-related cancer disease.

## 1. Introduction

Epidermal growth factor (EGF) or ErbB receptors belong to subclass I of the receptor tyrosine kinase protein’s family that consists of EGFR (ErbB1), HER2 (ErbB2, HER2/neu), HER3 (ErbB3), and HER4 (ErbB4) [1]. The three-dimensional structure of the EGFR is built up of three domains, namely; extracellular ligand binding domain region, transmembrane domain and cytoplasmic or an intracellular kinase domain [2]. Currently, there are two common classes of EGFR inhibitors, including monoclonal antibodies (mAbs) targeting the extracellular domain of EGFR, such as cetuximab (Erbitux), and small-molecule tyrosine kinase inhibitors (TKIs) targeting receptor’s catalytic domain of EGFR, such as gefitinib (Iressa^®^) and erlotinib (Tarceva^®^) [3,4,5]. EGFR-directed TKIs have the following mechanism: Upon binding of a specific ligand to EGFR’s binding domain, dimerization will occur to form heterodimeric receptor. This will activate the receptor’s autophosphorylation through the cytoplasmic tyrosine kinase catalytic domain. This catalytic activity initiates downstream regulation of many receptors’ signaling pathways, which are responsible for several critical processes including cell proliferation and differentiation, tissue homeostasis and tumorigenesis. Correspondingly, this means that they are responsible for cancer cell proliferation, arresting of the apoptosis process and stimulation of metastasis. On the other hand, TKIs which are highly selective for EGFR tyrosine kinase can inhibit autophosphorylation in a variety of EGFR-expressing human cancer cell lines. This inhibition takes place by competing with adenosine triphosphate (ATP) for its binding site on the intracellular domain of EGFR [6,7,8,9,10,11]. Thus, the development of small molecular compounds to inhibit EGFR is an important therapeutic approach for treating variety of cancers. Therefore, small molecule-molecule inhibitors that compete with either the ligand-binding domain or ATP binding pocket of the cytoplasmic tyrosine kinase domain can act as anticancer drugs.

Several small molecules based on quinazoline derivatives—gefitinib, erlotinib, lapatinib (Tykerb^®^, also known as GW-572016) and vandetanib (Zactima^TM^)—were recently approved for the treatment of breast cancer and non-small cell lung cancer (NSCLC) [9,10,12,13,14,15,16,17,18,19]. Although the therapeutic effect of the current anticancer quinazoline-based agents on different cancers have been well established, many side effects such as diarrhea, skin rashes, nausea, vomiting, hemorrhage and abnormal liver functions were also reported [17,20,21].

**Figure 1:**
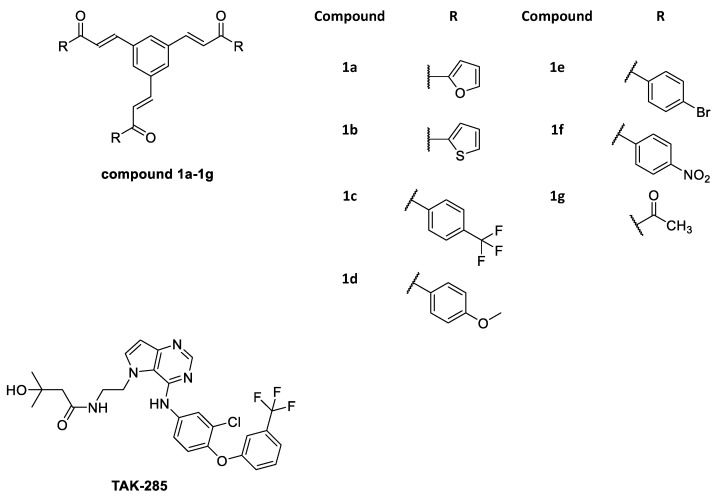
2D Structures of TAK-285 and novel chalcone derivatives **1a**–**1g**.

Clearly, as an anticancer agent it is necessary to find drugs with minimum adverse effects those provide more hope for patients. Hence, the use of chalcone derivatives was considered for minimizing unwanted side effects [22,23,24,25]. In addition, several studies revealed the ability of chalcone derivatives to become an important antimicrobial, antifungal, anti-mycobacterial, antimalarial, antiviral, anti-inflammatory, antioxidant, antileishmanial anti-tumor, and anticancer agents [26,27,28]. Thus, in this study chalcones have been used as EGFR inhibitors [26,27,28,29,30,31]. As a result, novel chalcone derivatives **1a**–**1g** (as shown in Figure 1) along with TAK-285, a known inhibitor co-crystallized with EGFR, have been proposed to be studied through computational docking and molecular dynamics (MD) techniques. This proposition was assumed to examine the binding interactions and binding energies within EGFR active site, expecting it to provide useful insights for designing effective drugs to treat EGFR-related cancers.

## 2. Materials and Methodology

### 2.1. Overview

The use of computational modelling methods helps to increase the efficiency of the drug discovery process as well as to reduce the experimental cost and time [32,33,34]. EGFR tyrosine kinase was selected as a therapeutic target for novel chalcone derivatives since it is a known and validated anticancer drug target. The X-ray crystallographic structure of EGFR kinase domain (PDB ID: 3POZ) with a resolution of 1.5 Å was selected from Protein Data Bank (www.rcsb.org/pdb) [35]. AutoDock 4.2 (The Scripps Research Institute, San Diego, CA, USA) was used to study the intermolecular interactions and binding energies of the proposed compounds in order to select compounds for further investigation by MD simulation using AMBER 14 (University of California, San Francisco, CA, USA) [36].

### 2.2. Software

The following software packages were used in the present research:

a) ACD/ChemSketch v. 2016.1.1 (www.acdlabs.com);
b) AutoDock 4.2 [37,38];
c) AMBER 14 [36].

### 2.3. Molecular Docking

All chalcone derivatives in Figure 1 have been drawn and saved as mol2 files by ChemSketch software and then converted to pdb files. Ligand files in pdb format were prepared by AutoDockTools. Once opened, charges were added, and all hydrogen atoms were merged. Molecular docking simulations of compounds **1a**–**1g** and TAK-285 (crystal structure) were performed against 3POZ utilizing AutoDock 4.2. Both atomic charges were added, and hydrogen atoms were merged to new chalcone derivatives and their targeted protein. Kollman and Gasteiger charges were added to protein and chalcone derivatives respectively. A set of grid maps were created, using AutoGrid 4 (The Scripps Research Institute, San Diego, CA, USA). A grid box was then utilized to select which area of the protein structure to be mapped. The box size was set to 22.5, 22.5 and 22.5 Å (x, y and z, respectively). Lamarckian genetic algorithm (LGA) was applied for energy optimization and minimization during docking simulation.

### 2.4. Molecular Dynamics

In this work two ligands, **1a** an **1d**, were chosen according to the results obtained from AutoDock 4.2 for further investigation by MD simulation along with TAK-285 using AMBER 14 [36]. Throughout a timescale of seven nanosecond (7 ns) for each system, these simulations were performed to study the key interactions in the protein’s active site.

#### 2.4.1. Model Setup

Three systems were prepared using crystal PDB structure (PDB ID: 3POZ). All hydrogen atoms were added explicitly by LEAP module in AMBER 14 package [36]. Amber ff14SB [39] force field was utilized for amino acids residues. General Amber force field (GAFF) [40] was used to describe the ligands. Also, sodium counter ions were added to the most negative positions of the prepared complexes to neutralize the systems [41]. the prepared complexes were immersed in TIP3P water box [42].

#### 2.4.2. Minimization

Each system minimization was initiated with 1000 steps of steepest decent method. That was followed by 1000 steps of conjugate gradient. Afterward, each minimized system was hydrated in a 10 Å truncated box of TIP3P water [42] and seven sodium ions were added for system charge neutralization. Each solvated complex was minimized under the same aforementioned conditions.

#### 2.4.3. Equilibration

Twenty picoseconds (ps) equilibration was performed for each system under full isotropic NVT condition at 310 K. The aim of this step was to enable the system to evolve from the starting configuration to reach equilibrium. Once equilibration was reached at 310 K, NPT condition was switched to allow the system to adjust its density continuously and naturally (20 ps). SHAKE algorithm was used to constrain all hydrogens in the system and the non-bonded interactions pair-list was generated with a cut-off distance of 12 Å. Equilibration was allowed up to 1 ns per simulation.

#### 2.4.4. Production Stage

Each simulation was performed for seven nanoseconds with constant pressure (NPT). The Berendsen barostat method was employed to control the pressure of both systems at 1 bar with an isotropic position scaling [43]. Furthermore, the temperature for each system was maintained at 310 K utilizing the Langevin thermostat method [44].

#### 2.4.5. MM-GBSA Calculation

The MMPBSA.py module of AMBER 14 and AmberTools 14 [36] were utilized to calculate free energy components of each system. This script automatically performs all the necessary steps to estimate the binding free energy of protein-ligand complexes utilizing the MM-PBSA method. The molecular-mechanical energy contributions were calculated by pmemd integrated within the AMBER software according to the force field where the topology files were created. By the following equations, the overall energy of the binding (ΔGbind) was calculated:

ΔGbind = ΔH − TΔS ≈ ΔEMM + ΔGsol − TΔS(1)

ΔEMM = ΔEint + ΔEelecis + ΔEvdw(2)

ΔGsolv = ΔGGB + ΔGSA(3)

where, ΔH is the enthalpy, T is the temperature in Kelvin and S is the entropy. ΔEMM describes the molecular mechanical (MM)energy change in the gas phase Which equals the sum of the following energies the internal energy ΔEint, the coulomb electrostatic term ΔEelecis and the vander Waals interaction term ΔEvdw respectively. ΔGsolv is the solvation free energy, ΔGGB is the electrostatic solvation energy (polar contribution) computed by both GB model and ΔGSA which describes the non-electrostatic solvation component (nonpolar contribution).

## 3. Results and Discussion

All results were obtained from two molecular modelling techniques; molecular docking utilizing AutoDock 4.2 and molecular dynamics simulations using AMBER 14.

### 3.1. Molecular Docking

Currently, AutoDock 4.2 was reported to be the most popular docking program. Its high accuracy and versatility had expanded its application [45,46]. Proposed chalcone derivatives were successfully docked against the 3POZ crystal structure, and the results are shown in Table 1. Based on the results of dockings, most of the proposed chalcone derivatives have shown good binding energies ranges (−9.36)–(−5.66) kcal/mol, as shown in Table 1. Compound **1f** revealed the lowest binding energy with −9.36 kcal/mol, while, **1g** and TAK-285 showed −5.66 and −5.85 kcal/mol, respectively. It is noteworthy that TAK-285 binding energy value is consistent with previous studies [47].

**Figure 2:**
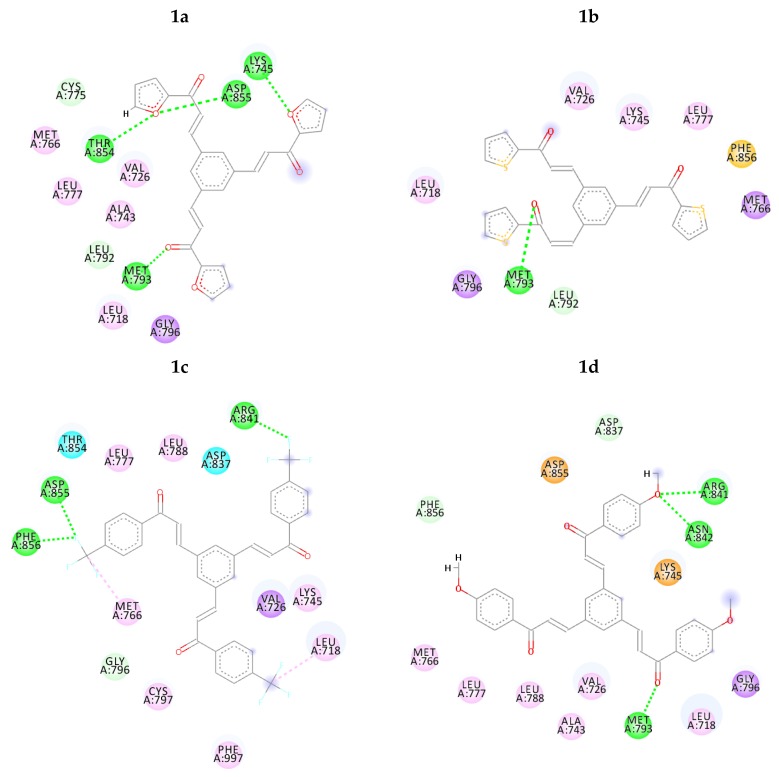

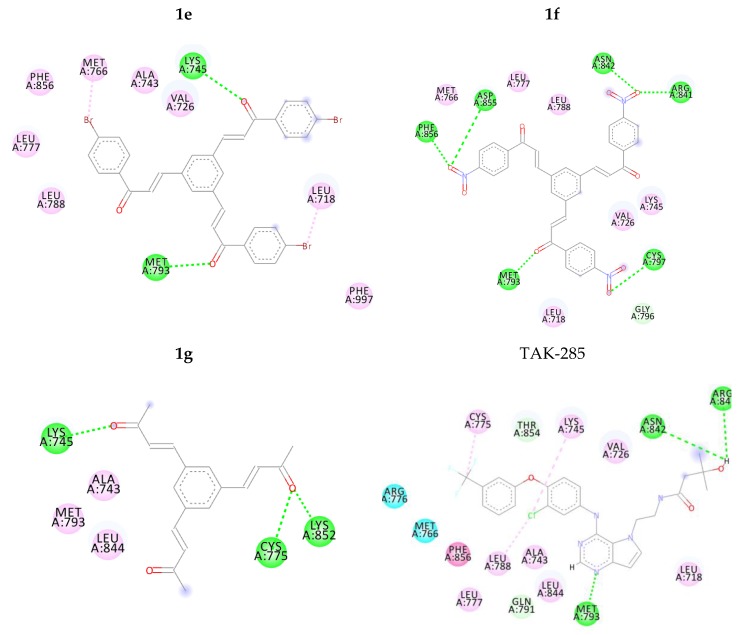
2D intermolecular interactions between docked compounds (**1a**–**1g** and TAK-285) and 3POZ protein. Green and Pink colored amino acids represent their contribution in hydrogen bond and hydrophobic interactions, respectively.

The intermolecular interactions of the docked compounds are displayed in Figure 2. TAK-285 binds within ATP binding pocket of the catalytic tyrosine kinase domain competing with ATP. The mechanism of inhibition is thought to be due to direct hydrogen bond interaction between the pyrimidine ring nitrogen and MET 93 [35], which can be seen in all proposed chalcone derivatives except **1c** and **1g**. Additionally, several amino acids performed conventional hydrogen bonds with TAK-285 namely, ARG 41 and ASN 42, those match compound **1d** hydrogen bond interactions. On the other hand, compounds **1a**, **1b**, **1e** and **1f** have made 4, 1, 2 and 6 hydrogen bond interactions, respectively, as shown in Table 1 and Figure 2.

Clearly, compound **1f** performed better hydrogen bond interactions retaining the important amino acids MET 93, ARG 41 and ASN 42. However, it was not selected for further investigation due to possible environmental toxicity, carcinogenicity and mutagenicity of aromatic nitro compounds [48].

**Figure 3:**
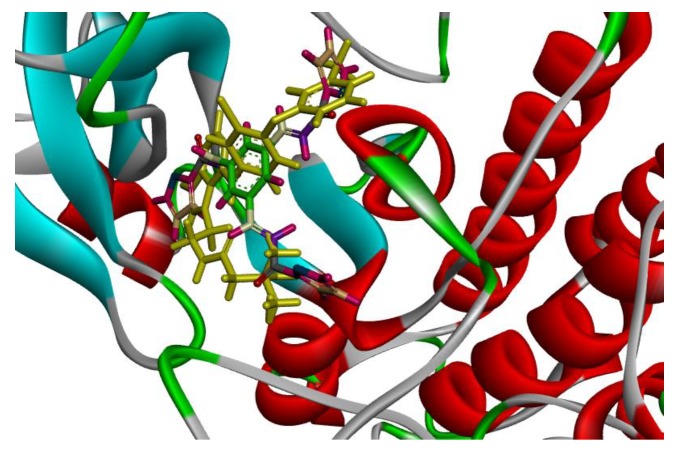
Solid ribbon representation of 3POZ docked with crystal structure of TAK-285 (yellow color) and inhibitor **1a** (colored) in the active site.

**Figure 4:**
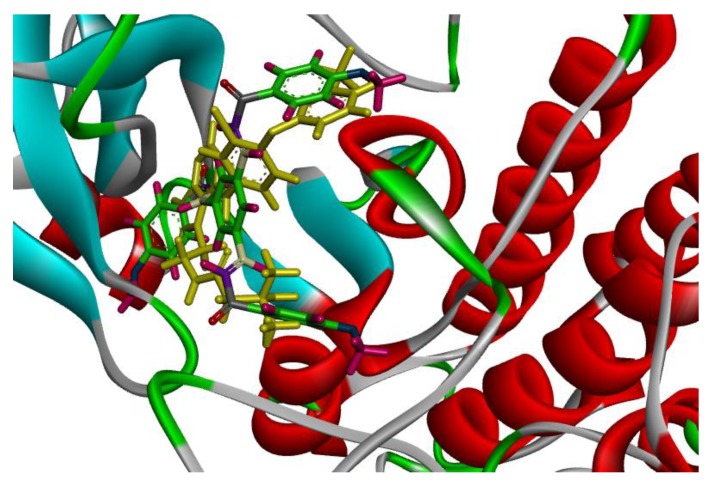
Solid ribbon representation of 3POZ docked with the crystal structure of TAK-285 (yellow color) and inhibitor **1d** (colored) in the active site.

On the other hand, compounds **1a** and **1d** were nicely bound in the active site forming several binding interactions with amino acids such as MET 93, THR 54, ASN 42, ARG 41, ASP 55, PHE 56, MET 66, LYS 45 including non-polar residues i.e., LEU 18, VAL 26 and PHE 56. These interactions match previous reported results by Subrahmanyam et.al. [49]. Therefore, it was decided to perform molecular dynamics simulation on compounds **1a** and **1d**, as well as, TAK-285 as a standard. Both compounds, along with TAK-285, showed similar binding positions in the active site as presented in Figure 3 and Figure 4.

### 3.2. Molecular Dynamics

According to molecular docking results, two compounds, **1a** and **1d**, as well as TAK-285 were selected to further investigate the structural changes upon ligand binding and intermolecular interactions using molecular dynamics (MD) simulations. Seven nanoseconds molecular dynamics simulation have been carried out for each of the three systems and the last nanosecond was used to calculate the free energy of ligand binding using MM-GBSA method. The stability of the systems was examined by monitoring the thermodynamic properties, such as pressure, temperature, potential energy and kinetic energy, as shown in Figures S1–S3 in the Supplementary Materials.

#### 3.2.1. Root Mean Square Deviation (RMSD)

**Figure 5:**
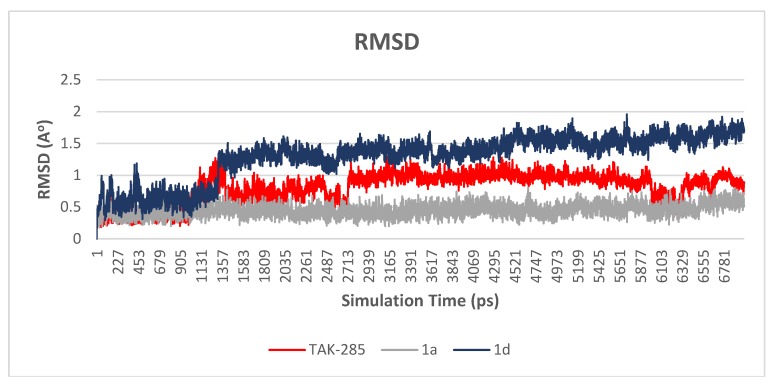
Plot of RMSD vs. time graph for TAK-285 (red), **1a** (gray) and **1d** (blue). Compound **1a** shown to be fluctuating around 0.5 Å, while compounds **1d** and TAK-285 were fluctuating around 1.4 and 1.0 Å.

**Figure 6:**
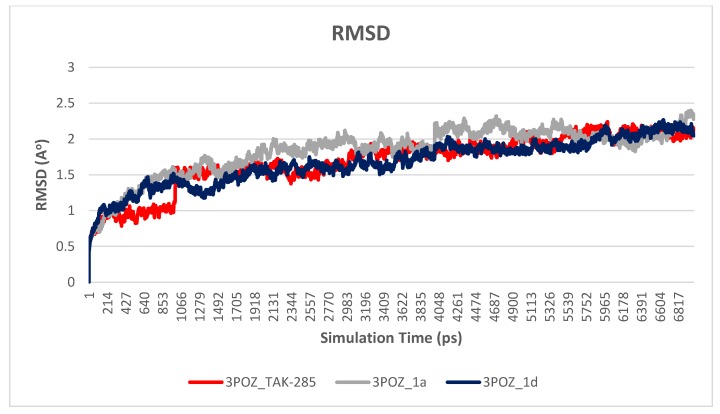
Plot of RMSD vs. time graph for 3POZ-TAK-285 (red), 3POZ-**1a** (gray) and 3POZ-**1d** (blue), with an average RMSD values of 1.7, 1.8, and 1.7 Å, respectively.

The main purpose of the MD studies was to investigate the positional and conformational changes of inhibitor upon binding to the active site which provides an insight of the binding stability. For the ease of comparison, the RMSD differences between the systems were evaluated for the ligands and proteins separately. A plot of RMSD through time of crystal structure of TAK-285 and compounds **1a** and **1d** is presented in Figure 5. RMSD analysis showed that compound **1a** has the lowest RMSD value (0.5 Å), while TAK-285 and compound **1d** were 1.0 Å and 1.4 Å, respectively. Fluctuation of compound **1a** is lower than compounds **1d** and TAK-285 within the active site. On the other hand, the RMSD analysis of the three systems were very close to each other with 1.7, 1.8, and 1.7 Å for 3POZ-TAK-285, 3POZ-**1a**, and 3POZ-**1d**, respectively as presented in Figure 6. It is worth to note that 3POZ crystal structure obtained from PDB is in the inhibited conformation since it is crystalized with TAK-285 inhibitor. All the three protein-ligand trajectories exhibit low backbone RMSD values, indicating the EGFR inhibitor complexes are quite stable, which added an extra credibility of the docking results.

#### 3.2.2. Hydrogen Bonding Analysis

Hydrogen bonds formed between the protein and the ligands were mostly seen in the activation loop region of the protein during the simulation. Thus, they may play an essential role in stabilizing protein ligand complexes [50]. In this study, the hydrogen bonds that presents more than 80% during the simulation will be considered as “strong hydrogen bonds”. Despite that, “medium hydrogen bonds” are the hydrogen bonding that existed between 50–80% of the simulation time, while hydrogen bonds that appear in 10–50% of the simulation time will be assigned as “weak hydrogen bonds” [51].

**Figure 7:**
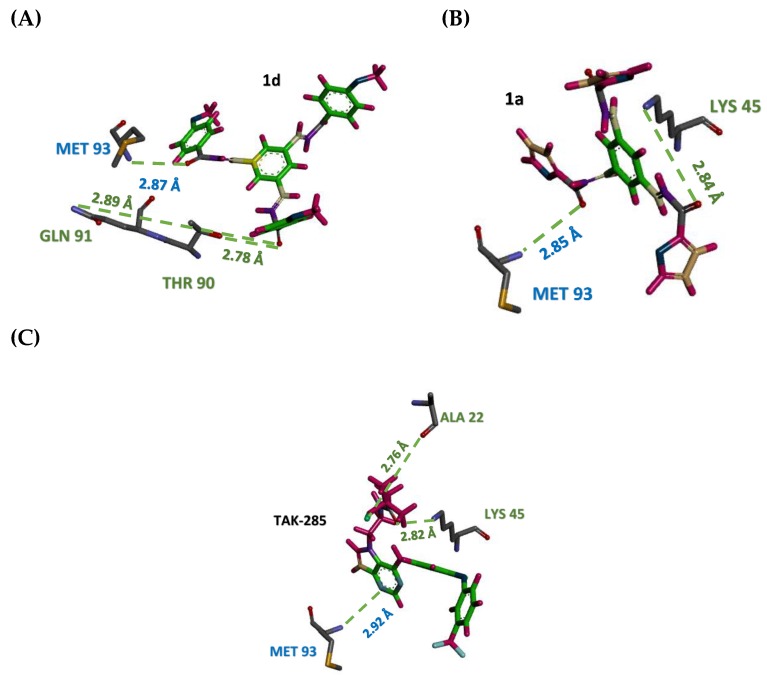
Stick representation of simulated compounds **1a** (**A**), **1d** (**B**) and TAK-285 (**C**) site forming hydrogen bond (green dots) interactions with amino acid residues in the active. MET 93 amino acid found to participate in the hydrogen bond interaction with the three simulated compounds.

Selvaraj et. al. [52] revealed the importance of the presence of hydrogen bonds between EGFR active site amino acid MET 93 and the hetero atom (nitrogen) within the inhibitor. In addition, amino acid ASP 55 and LYS 45 were involved in the interaction. The same study found that dual inhibitor TAK-285 binds with the ATP binding pocket of EGFR competing with ATP. They concluded that the inhibition is due to direct hydrogen bond formation between pyrimidine ring nitrogen and MET 93, which was found to be identical during the 7 ns trajectory in this study, thus enhancing the accuracy of our results as presented in Table 2 and shown in Figure 7.

Upon extensive hydrogen bond analysis throughout 7ns simulation, TAK-285 was found to form hydrogen bonds with MET 93 in 35.2% occurrence frequency, while it was 80.74% and 65.43% for **1a** and **1d**, respectively. These results indicate the ability of the selected compounds to retain essential hydrogen bonds for inhibition activity more than TAK-285.

Both TAK-285 and **1a** formed more than one hydrogen bond with LYS 45, with the occurrence frequency of 12.67% for TAK-285 and 4.54% for **1a**, while it is absent in compound **1d**. Additionally, ASP 55 was found to form one hydrogen bond with TAK-285, **1d** and two hydrogen bonds with **1a**, with very low occupancy. On the other hand, compound **1d** shown to perform one hydrogen bond with THR 90, with 52.83% which strengthen the interaction. Moreover, GLN 91 shown to perform hydrogen bond interaction only with inhibitor **1d** with 8.69% occurrence frequency. Furthermore, the results of molecular dynamic study have also shown that proposed chalcone derivatives **1a** and **1d** may have the ability to steadily anchor to kinase domain of EGFR to exert an inhibitory effect.

#### 3.2.3. Free Energy of Binding Calculation

The overall objective of the MM-PBSA method and its complementary MM-GBSA method is to calculate the free energy difference between two states which most often represent the bound and unbound state of two solvated molecules or alternatively to compare the free energy of two different solvated conformations of the same molecule [53].

As shown in Table 3, Van der Waals and electrostatic energy values (intermolecular interaction) performed by compound **1a** were much higher than TAK-285 and **1d**. This may indicate the ability of TAK-285 and **1d** to have higher affinity against the receptor than compound **1a**. Accordingly, these differences in energies could be related to the higher number of hydrogen bonds 4 and 3 formed by (TAK-285 and **1d**) respectively in comparison to inhibitor **1a** (2 hydrogen bonds) as revealed in Section 3.2.3. Although the other energy component values seem close to each other, a significant difference between the three ligands can be found in the electrostatic solvation energy (polar contribution) calculated by GB model (ΔGGB). Summation of the energies showed that inhibitor **1d** had better binding energy, −56.62 kcal/mol, in comparison to **1a** (−44.05 kcal/mol).

## 4. Conclusions

According to docking results, the proposed chalcone derivatives show good to moderate docking energies that range from −9.36 to −5.66 kcal/mol, as stated in Table 1. After a careful analysis of intermolecular interactions and docking energies for each compound, it was found that compounds **1a** and **1d** were nicely bound within the active site. These are compounds shown to match the co-crystallised inhibitor (TAK-285) intermolecular interactions with MET 93, ARG 41 and ASN 42. Both compounds displayed interaction with MET 93 which thought to be responsible for inhibition mechanism according to previous studies. Moreover, compound **1d** showed similar interactions with TAK-285 by interacting with ARG 41 and ASN 42 amino acids, as shown in Figure 2. Thus, it was suggested to proceed with compounds **1a** and **1d** for molecular dynamics simulation study.

Molecular dynamics simulations showed that the three simulated systems exhibited close RMSD values to each other with 1.7, 1.8, and 1.7 Å for 3POZ-TAK-285, 3POZ-**1a**, and 3POZ-**1d**, respectively. Moreover, the RMSD values shown low values, indicating good stability that may strengthen the reliability of the docking results. Additionally, extensive hydrogen bond analysis throughout 7 ns simulation revealed the ability of the proposed ligands to retain the essential interactions with MET 93, LYS 45 and THR 90 amino acids. Still, the results of molecular dynamic study have also shown that proposed chalcone derivatives **1a** and **1d** may have the ability to steadily anchor to kinase domain of EGFR to exert an inhibitory effect.

Finally, MM-GBSA calculations highlight on the capability of the ligands to bind strongly within the active site with binding energies of −44.04 and −56.6 kcal/mol for compounds **1a** and **1d**, respectively. Compound **1d** was shown to have a close binding energy with TAK-285 (−66.17 kcal/mol), which indicates a high chance for compound **1d** to exhibit an inhibition activity, thus to recommend synthesising it and perform biological activity studies.

## Figures and Tables

**Table 1:** The lowest binding energies obtained from AutoDock 4.2 and interacting amino acids.

Compounds	Lowest Binding Energy (kcal/mol)	Interacting Amino Acids
TAK-285	−5.85	MET 93, ARG 41, ASN 42, LYS 45, LEU88, CYS 75
**1a**	−8.49	MET 93, LYS 45, ASP 55, THR 54
**1b**	−8.82	MET 93
**1c**	−7.13	ARG 41, ASP 55, PHE 56, MET 66, LEU 18
**1d**	−8.63	MET 93, ARG 41, ASN 42
**1e**	−8.68	MET 93, LYS 45, MET 66, LEU 18
**1f**	−9.36	MET 93, ARG 41, ASN 42, ASP 55, PHE 56, CYS 97
**1g**	−5.66	CYS 75, LYS 45, LYS 52

**Table 2:** Hydrogen Bonds Analysis with MD Simulation for the EGFR Inhibitors within the ATP Active Site.

Inhibitor	H-Bond Acceptor (Atom@res)	DonorH	Donor	Percentage Occupancy (%)	Average Distance (Angstrom)	Average Angle (Degree)
TAK-285	03P 318@N	MET 93@H	MET 93@N	35.2	2.9236	163.05
	03P 318@O	LYS 45@HZ2	LYS 45@NZ	12.67	2.8211	160.01
	ALA 22@O	03P 318@H22	03P 318@O2	10.54	2.7619	160.63
	SER 20@O	03P 318@H22	03P 318@O2	3.89	2.808	163.49
**1a**	L1A 318@O4	MET 93@H	MET 93@N	80.74	2.8447	161.96
	L1A 318@O	LYS 45@HZ2	LYS 45@NZ	4.54	2.8384	153.83
**1d**	L1D 318@O4	MET 93@H	MET 93@N	65.43	2.8655	161.95
	L1D 318@O2	THR 90@HG1	THR 90@OG1	52.83	2.7757	160.93
	L1D 318@O2	GLN 91@HE21	GLN 91@NE2	8.69	2.8898	153.39

**Table 3:** Total binding energy and its components of TAK-285, **1a** and **1d** complexes obtained from MM-GBSA.

Energy Component	TAK-285	1a	1d
VDWAALS	−77.1403	−60.4638	−68.1365
EEL	−24.8001	−20.18	−25.2256
ΔG_gas_ (vdw + EEL)	−101.9404	−80.6438	−93.3621
E_GB_	44.935	44.9765	45.7953
E_SURF_	−9.173	−8.3796	−9.0521
ΔG_solv_ (E_GB_ +E_SURF_)	35.762	36.5969	36.7432
ΔG_MMGBSA_ (ΔGgas +ΔGsolv)	−66.1784	−44.0469	−56.6189

## Supplementary Materials

The following are available online, Figure S1: MD simulation: Temperature profile during 7 ns for the (**a**) TAK-285, (**b**) **1a**, (**c**) **1d**; Figure S2: MD simulation: pressure profile during equilibration for the (**a**) TAK-285, (**b**) **1a**, (**c**) **1d**; Figure S3: MD simulation: Energy profile for the (**a**) TAK-285, (**b**) **1a**, (**c**) **1d**, total energy (grey color), potential energy (red color), kinetic energy (green color).
