# Supplementary material for: Structure-Based Drug Design Studies Toward the Discovery of Novel Chalcone Derivatives as Potential Epidermal Growth Factor Receptor (EGFR) Inhibitors
Source: Molecules. 2018 Dec 5;23(12):3203. doi: 10.3390/molecules23123203 (PMC6321200; doi:10.3390/molecules23123203)
Supplement: Supplementary file 1 [file molecules-23-03203-s001.pdf]

Supplementary Materials

(a)

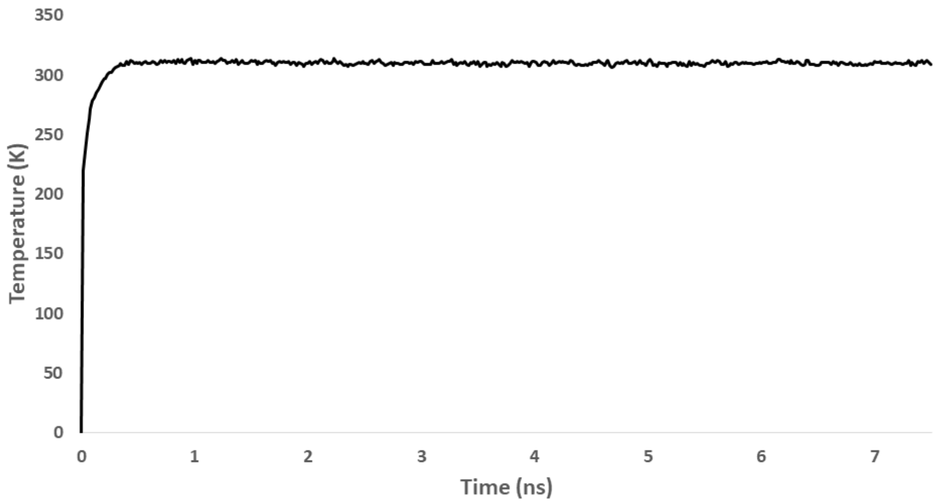

(b)

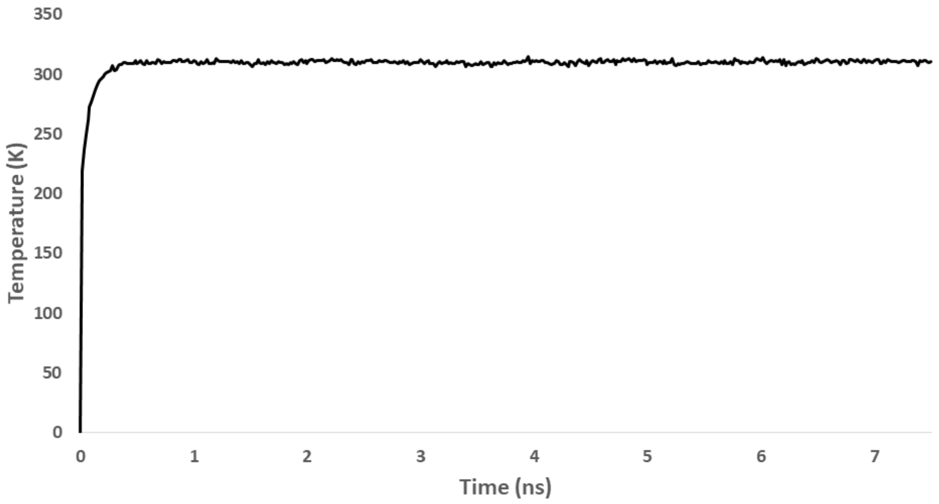

(c)

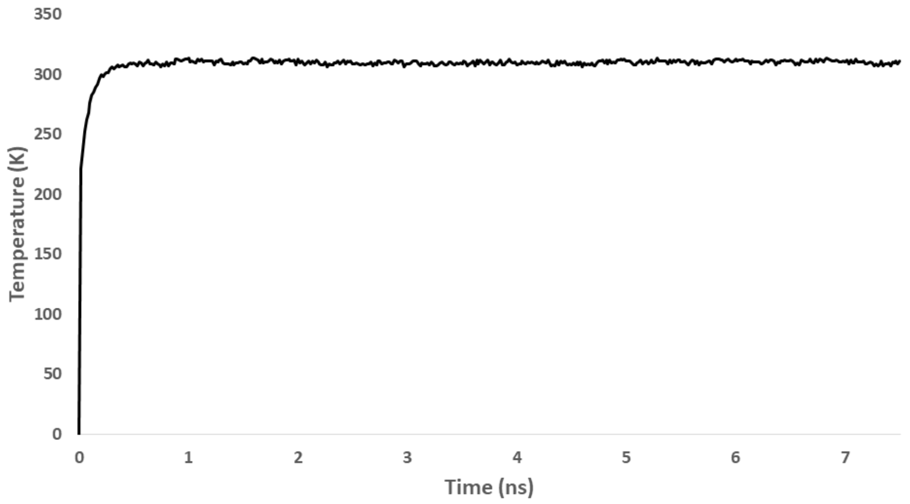

Figure S1: MD simulation: Temperature profile during 7 ns for the **(a) TAK-285**, **(b) 1a**, **(c) 1d**.

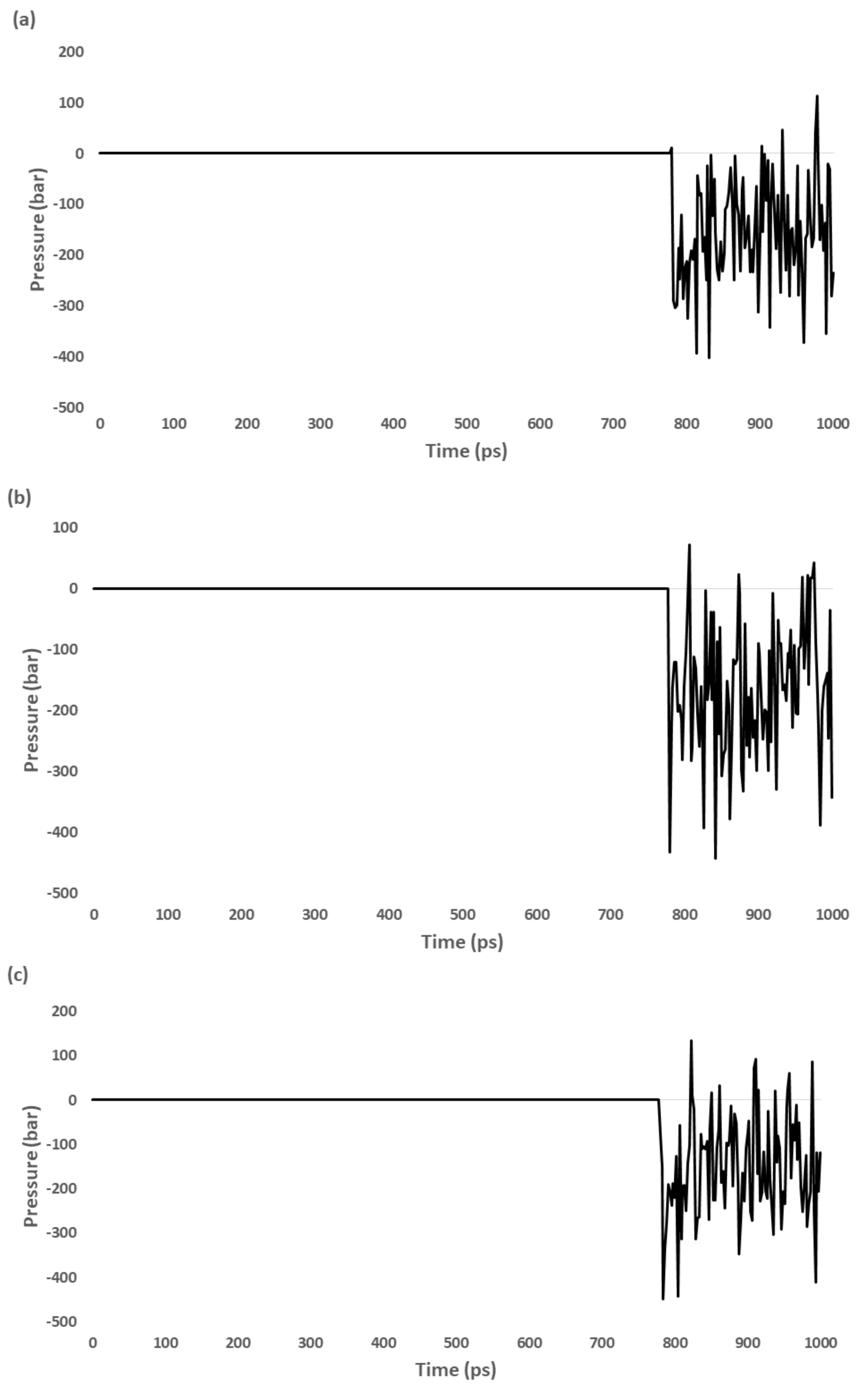

Figure S2: MD simulation: pressure profile during equilibration for the **(a) TAK-285**, **(b) 1a**, **(c) 1d**.

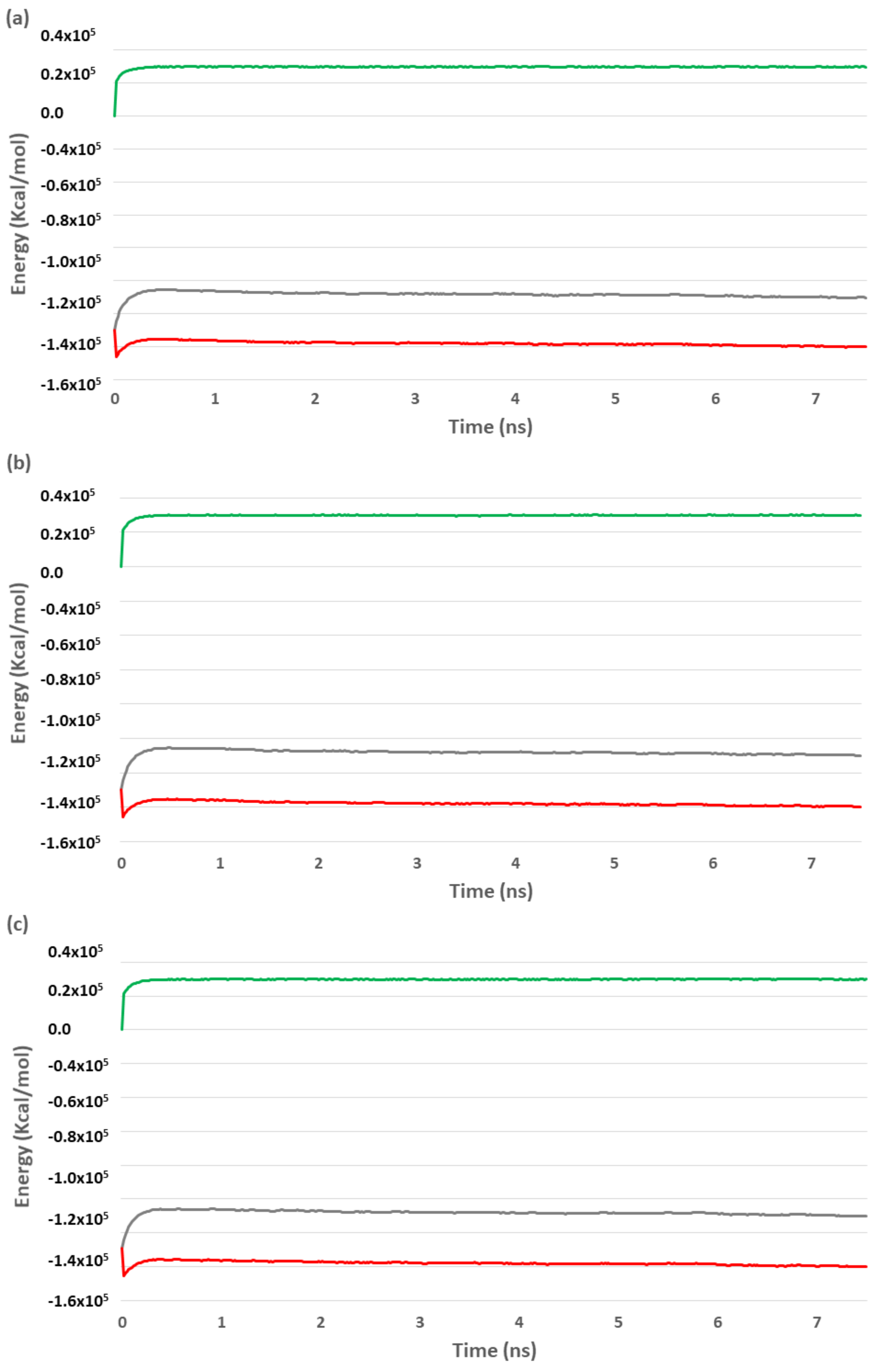

Figure S3. MD simulation: Energy profile for the **(a) TAK-285**, **(b) 1a**, **(c) 1d**. total energy (grey color), potential energy (red color), kinetic energy (green color)
